# Combining multimodal connectivity information improves modelling of pathology spread in Alzheimer’s disease

**DOI:** 10.1162/imag_a_00089

**Published:** 2024-02-05

**Authors:** Elinor Thompson, Anna Schroder, Tiantian He, Cameron Shand, Sonja Soskic, Neil P. Oxtoby, Frederik Barkhof, Daniel C. Alexander, for the Alzheimer’s Disease Neuroimaging Initiative

**Affiliations:** UCL Centre for Medical Image Computing, Department of Computer Science, University College London, London, United Kingdom; UCL Centre for Medical Image Computing, Department of Medical Physics and Biomedical Engineering, University College London, London, United Kingdom; Department of Radiology & Nuclear Medicine, Amsterdam UMC, Vrije Universiteit, the Netherlands; UCL Queen Square Institute of Neurology, University College London, London, United Kingdom

**Keywords:** Brain connectivity, Alzheimer’s disease, network spreading models

## Abstract

Cortical atrophy and aggregates of misfolded tau proteins are key hallmarks of Alzheimer’s disease. Computational models that simulate the propagation of pathogens between connected brain regions have been used to elucidate mechanistic information about the spread of these disease biomarkers, such as disease epicentres and spreading rates. However, the connectomes that are used as substrates for these models are known to contain modality-specific false positive and false negative connections, influenced by the biases inherent to the different methods for estimating connections in the brain. In this work, we compare five types of connectomes for modelling both tau and atrophy patterns with the network diffusion model, which are validated against tau PET and structural MRI data from individuals with either mild cognitive impairment or dementia. We then test the hypothesis that a joint connectome, with combined information from different modalities, provides an improved substrate for the model. We find that a combination of multimodal information helps the model to capture observed patterns of tau deposition and atrophy better than any single modality. This is validated with data from independent datasets. Overall, our findings suggest that combining connectivity measures into a single connectome can mitigate some of the biases inherent to each modality and facilitate more accurate models of pathology spread, thus aiding our ability to understand disease mechanisms, and providing insight into the complementary information contained in different measures of brain connectivity

## 1 Introduction

Alzheimer’s disease (AD) is a debilitating neurodegenerative disease that affects around 25 million people worldwide (Qiu et al., 2009). A key pathology in AD is the accumulation of hyperphosphorylated tau protein aggregates in the brain. Tau burden is closely linked to grey matter atrophy, as measured with MRI (La Joie et al., 2020; Ossenkoppele et al., 2016), both of which are associated with cognitive decline (Querbes et al., 2009). Post-mortem histology has suggested a typical pattern of tau spread, beginning in the transentorhinal cortex and spreading to the hippocampus, followed by the limbic and association cortices, as described by the Braak staging system (Braak & Braak, 1991). In the past decade, the development of tau PET tracers has facilitated the mapping of this pathology *in vivo* (Chien et al., 2013). Atrophy spread follows a similar trajectory to that of tau, beginning in the medial temporal and fusiform regions, before spreading to the posterior temporal and parietal cortices, and eventually to the frontal cortex (Whitwell, 2010).

Computational models of tau and atrophy propagation have been used to probe the mechanisms underlying this process, often based on the “prion hypothesis” of transsynaptic spread. They typically model the self-propagation of misfolded proteins between connected brain regions. The first example of this approach was the network diffusion model, which models the diffusive propagation of pathology, mediated by the brain’s connectivity network (Raj et al., 2012). More complex models have since been developed that include additional biologically-relevant parameters such as accumulation, clearance, and propagation rates (Fornari et al., 2019; Garbarino & Lorenzi, 2021; Iturria-Medina et al., 2014; Vogel et al., 2020; Yang et al., 2021). More recent work has demonstrated the ability of these models to predict longitudinal tau progression in individuals (Schäfer et al., 2020; Yang et al., 2021).

The link between brain connectivity and the patterns of pathological changes was first demonstrated by Seeley et al. (2009), who showed that the distinct patterns of atrophy observed in patients with five different dementias corresponded to functional brain networks measured with functional MRI (fMRI). Building on this work, connectomes derived from functional connectivity have commonly been used as the basis of disease propagation models (Brown et al., 2019; Vogel et al., 2020; Zhou et al., 2012). Other studies have used structural white matter connectomes derived from MR tractography, which more directly model the substrate for transsynaptic spread (Fornari et al., 2019; Iturria-Medina et al., 2014; Raj et al., 2012, 2015; Schäfer et al., 2020; Vogel et al., 2020), or Euclidean distance matrices to model pathology spread based on spatial proximity (Vogel et al., 2020; Weickenmeier et al., 2019). In addition to functional connectivity and tractography, other techniques for estimating brain connectivity are typically based on the similarity of different measures, such as cortical thickness or microstructure, either between regions in an individual (Seidlitz et al., 2018; Tijms et al., 2012), or across individuals (Alexander-Bloch, Giedd, et al., 2013; He et al., 2007). Connectomes derived from these approaches have been shown to exhibit similar graph-theoretic properties to those from tractography. However, each method has its own drawbacks and methodological biases, which leads to modality-specific false positive and false negative findings. For example, it is well documented that tractography is prone to false positive “connections”, due to the ill-posed nature of the tracking process (Maier-Hein et al., 2017; Thomas et al., 2014). On the other hand, measurements of functional connectivity with fMRI are confounded by physiological noise in the BOLD signal (Krüger & Glover, 2001), and structural covariance networks lack comparability across different datasets (Carmon et al., 2020).

In this paper, we explore whether combining information from different measures of connectivity can help identify a more robust network that best supports models of neurodegeneration in AD. First, we compare the performance of five different connectome types for modelling tau and atrophy propagation with the network diffusion model. Using tau PET and structural MRI data from a cohort of individuals with dementia and mild cognitive impairment (MCI), we test each model’s ability to reproduce observed pathology patterns. This builds on previous work comparing structural, functional, and distance-based connectomes for modelling the spread of pathology in neurodegenerative disease (Raj & Powell, 2021; Vogel et al., 2020). We extend these studies by including the anatomically informed geodesic distance rather than Euclidean distance, as well as the morphological and microstructural covariance networks. We also test the performance across a range of thresholds and seed regions, and benchmark against modality-specific null distributions of rewired connectomes. However, our primary novel contribution is to combine the different connectivity networks to generate a connectome optimised for the modelling of neurodegenerative processes. This new, task-driven framework provides insight into the connections that facilitate disease spread and improves the ability of our model to predict patterns of pathology.

## 2 Methods

**Fig. 1.**
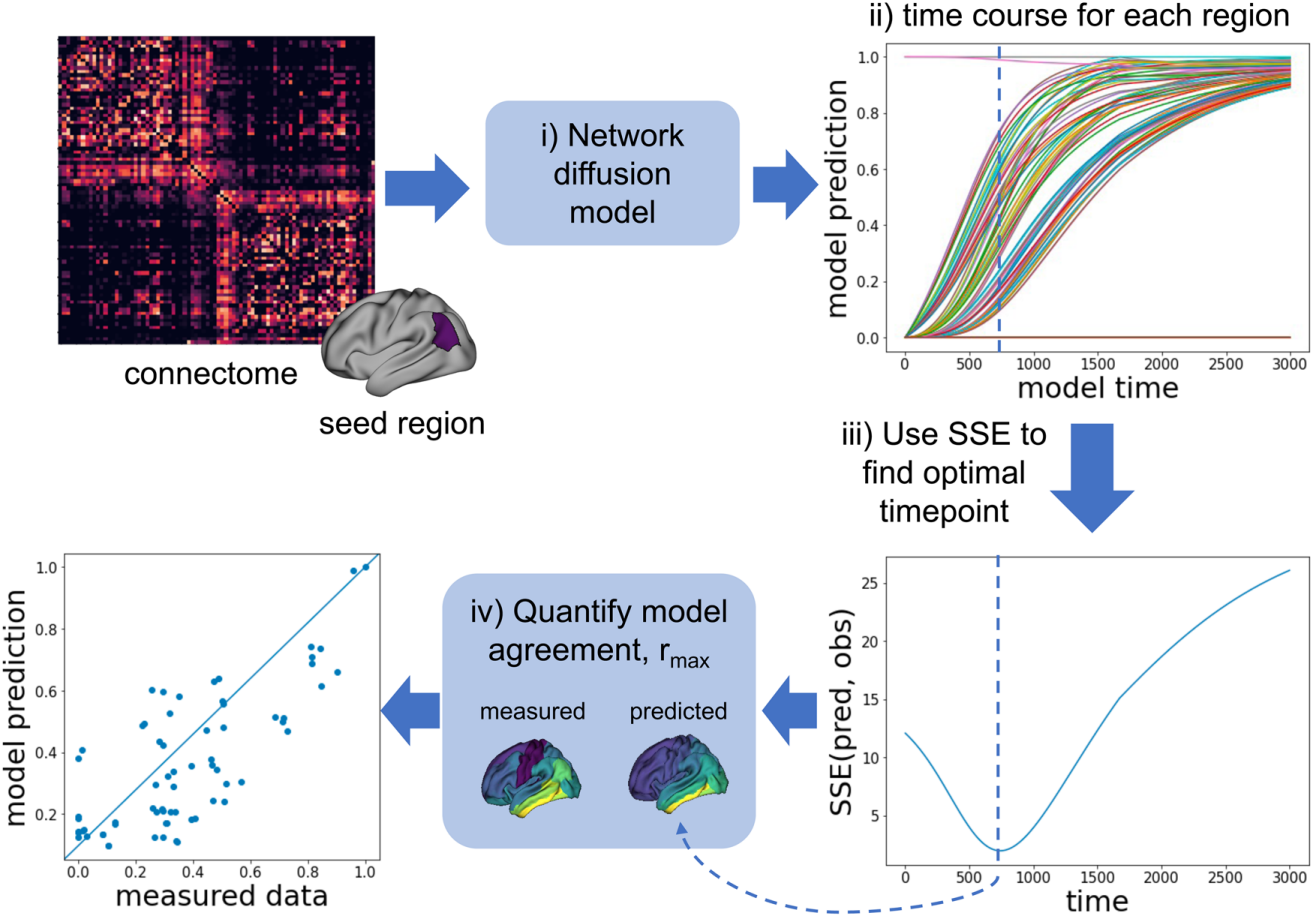
(i) The network diffusion model simulates pathology spread through the brain’s connectome, with a single seed region approximating the pathology pattern at t = 0. (ii) This yields a time course of predicted pathology for each brain region. (iii) Model time is arbitrary and so time is scanned to find the best model fit to the measured data, using sum of squared error (SSE). (iv) The Pearson’s correlation value (r_max_) between this model prediction and the observed data quantifies the goodness of the model fit.

Figure 1 shows an overview of the modelling approach. Connectomes derived from different brain measurements were used as inputs to the network diffusion model, in which pathology spread is modelled as a diffusive process between connected brain regions. The simulated disease patterns from the model were compared to measured data to find the model timepoint that best resembles the spatial distribution of *in vivo* tau or atrophy.

### 2.1 Data

We used data from the Alzheimer’s Disease Neuroimaging Initiative (ADNI), the Anti-Amyloid Treatment in Asymptomatic AD (A4) study, the Open Access Series of Imaging Studies (OASIS-3), and the Microstructure-Informed Connectomics (MICA-MICs) dataset, which all obtained institutional review board approvals for their respective studies. Informed consent was obtained from all participants for being included in these studies.

#### 2.1.1 PET data and pre-processing

We used flortaucipir tau PET data collected as part of ADNI (http://adni.loni.usc.edu). ADNI was launched in 2003 as a public-private partnership, led by Principal Investigator Michael W. Weiner, MD. The primary goal of ADNI has been to test whether serial MRI, PET, other biological markers, and clinical and neuropsychological assessment can be combined to measure the progression of MCI and early AD. For up-to-date information, see www.adni-info.org. Data were included from 134 individuals with a diagnosis of dementia or mild cognitive impairment, and amyloid-beta positive status (56 women; 73.19 ± 7.23 years). Amyloid-beta positivity was determined by applying a cut-off value of 1.11 to the summary florbetapir cortical standardised uptake value ratio (SUVR), normalised by whole cerebellum (Royse et al., 2021). Data from an additional group of 21 amyloid-positive, cognitively unimpaired individuals with elevated tau PET were used to test the performance of the model across disease stage. Due to their amyloid pathology, these individuals are in the AD continuum, but are cognitively unimpaired. To find individuals with elevated tau, we identified those with amyloid-positive status whose tau SUVR in the medial temporal region was two standard deviations above the mean of the amyloid-negative group, following the approach taken in Young et al. (2022). To avoid bias towards individuals, only the final tau PET timepoint was included from individuals with multiple scans. We used regional summary flortaucipir data from the ADNI database that had been pre-processed according to the ADNI Flortaucipir processing pipeline (https://adni.bitbucket.io/reference/docs/UCBERKELEYAV1451/UCBERKELEY_AV1451_Methods_Aug2018.pdf), which provides a mean tau PET intensity value for each of the regions in the Desikan-Killiany atlas (Desikan et al., 2006), without partial volume correction. Regional tau SUVRs were generated by intensity normalising using the inferior cerebellum as a reference region.

As an external validation set, we used flortaucipir PET data from the A4 Study (Sperling et al., 2014, 2020). The A4 Study was a secondary prevention trial in preclinical AD, aiming to test whether solanezumab could slow cognitive decline associated with brain amyloid accumulation in cognitively unimpaired older individuals. We used data from 126 amyloid-positive individuals with elevated tau levels (75 women; 72.68 ± 4.62 years), collected at baseline. Amyloid status was determined using a hybrid quantitative and qualitative method established by the A4 Study (Pontecorvo et al., 2017). SUVRs were calculated using the multi-platform software AmyPET (https://github.com/AMYPAD/AmyPET), an extension of NiftyPET (Markiewicz et al., 2018, 2021), with the cerebellar grey matter used as a reference region. Demographic information for all tau PET datasets can be found in Table 1. As off-target flortaucipir binding is known to affect sub-cortical regions in particular (Groot et al., 2022), only tau PET data from the 68 FreeSurfer cortical regions were used from both datasets. The SUVR values were min-max scaled onto the interval [0,1] to match the simulated data from the network diffusion model.

**Table 1.** Demographic information for the ADNI and A4 tau PET datasets.

	ADNI (cognitively impaired)	ADNI (cognitively unimpaired)	A4
Number of individuals, n	134	21	126
Age (years, mean ±std)	73.2 ± 7.2	72.9 ± 6.5	72.7 ± 4.6
Females, n (%)	56 (42%)	17 (81%)	75 (60%)
MMSE (mean score ± std)	24.7 ± 4.8	28.1 ± 2.2	28.4 ± 1.3
Diagnostic group, n (%)			
Cognitively normal	-	21 (100%)	126 (100%)
MCI	83 (62%)	-	-
Dementia	51 (38%)	-	-
Diagnosis – scan interval (days, mean [min, max])	39.8 [0, 247]	36.8 [1, 265]	-

#### 2.1.2 Structural MRI-derived atrophy measurements

MRI data were included from 450 individuals from ADNI with a diagnosis of dementia or MCI and amyloid-beta positive status (198 women; age at scan 73.54 ± 7.00 years). Detailed MRI protocols are provided on the ADNI protocol website: (http://adni.loni.usc.edu/methods/documents/mri-protocols). As with the tau PET scans, only the final timepoints were used.

As a validation dataset, we used structural MRI data from OASIS-3 (LaMontagne et al., 2019). OASIS is a retrospective compilation of multimodal data focussed on the effects of healthy aging and AD (http://www.oasis-brains.org/). Using the same inclusion criteria as the ADNI cognitively impaired group, we identified a group of 71 amyloid-beta positive individuals with MCI or dementia. MCI was defined as a clinical dementia rating (CDR) score of 0.5, and dementia as having a CDR score of 1 or above.

T1-weighted scans from both datasets were processed using FreeSurfer 7.1.1 (https://github.com/e-dads/freesurfer). Following normalisation of regional brain volumes by intracranial volume, subject-specific regional atrophy scores were estimated as z-scores with respect to age-matched healthy controls, as in Raj et al. (2015). The demographic information related to the disease groups and the controls are shown in Table 2. Atrophy scores were mapped onto the interval [0,1] using the logistic function. Only data from cortical regions were used, for consistency with the tau data.

**Table 2.** Demographic information for the ADNI and OASIS structural MRI datasets.

	ADNI	ADNI (controls)	OASIS-3	OASIS-3 (controls)
Number of individuals, n	450	777	71	657
Age (years, mean ±std)	73.5 ± 7.0	72.3 ± 6.4	75.5 ± 8.1	75.7 ± 5.0
Females, n (%)	198 (44%)	438 (56%)	34 (48%)	365 (56%)
MMSE (mean score ± std)	23.5 ± 5.4	28.8 ± 2.9	26.1 ± 3.2	28.9 ± 1.3
Diagnostic group, n (%)				
Cognitively normal	-	777 (100%)	-	657 (100%)
MCI	221 (49%)	-	21 (70%)	-
Dementia	229 (51%)	-	50 (30%)	-
Diagnosis – scan interval (days, mean [min, max])	3.5 [0, 134]	7.6 [0, 126]	65.1 [0, 191]	77.4 [0, 329]

The control groups were used to provide a reference for the atrophy measure in the cognitively impaired groups.

#### 2.1.3 Connectivity measurements

We used reference brain connectivity data from the MICA-MICs dataset (Royer et al., 2022). Data were collected on a 3 T Siemens Magnetom Prisma-Fit, equipped with a 64-channel head coil, at the Brain Imaging Centre of the Montreal Neurological Institute and Hospital from 50 healthy participants (23 women; aged 29.54 ± 5.62 years at scan). The dataset contains diffusion MRI, resting-state fMRI, and T1-weighted scans, including quantitative T1 relaxometry data. Acquisition parameters and pre-processing methods are detailed in Royer et al. (2022).

**Fig. 2.**
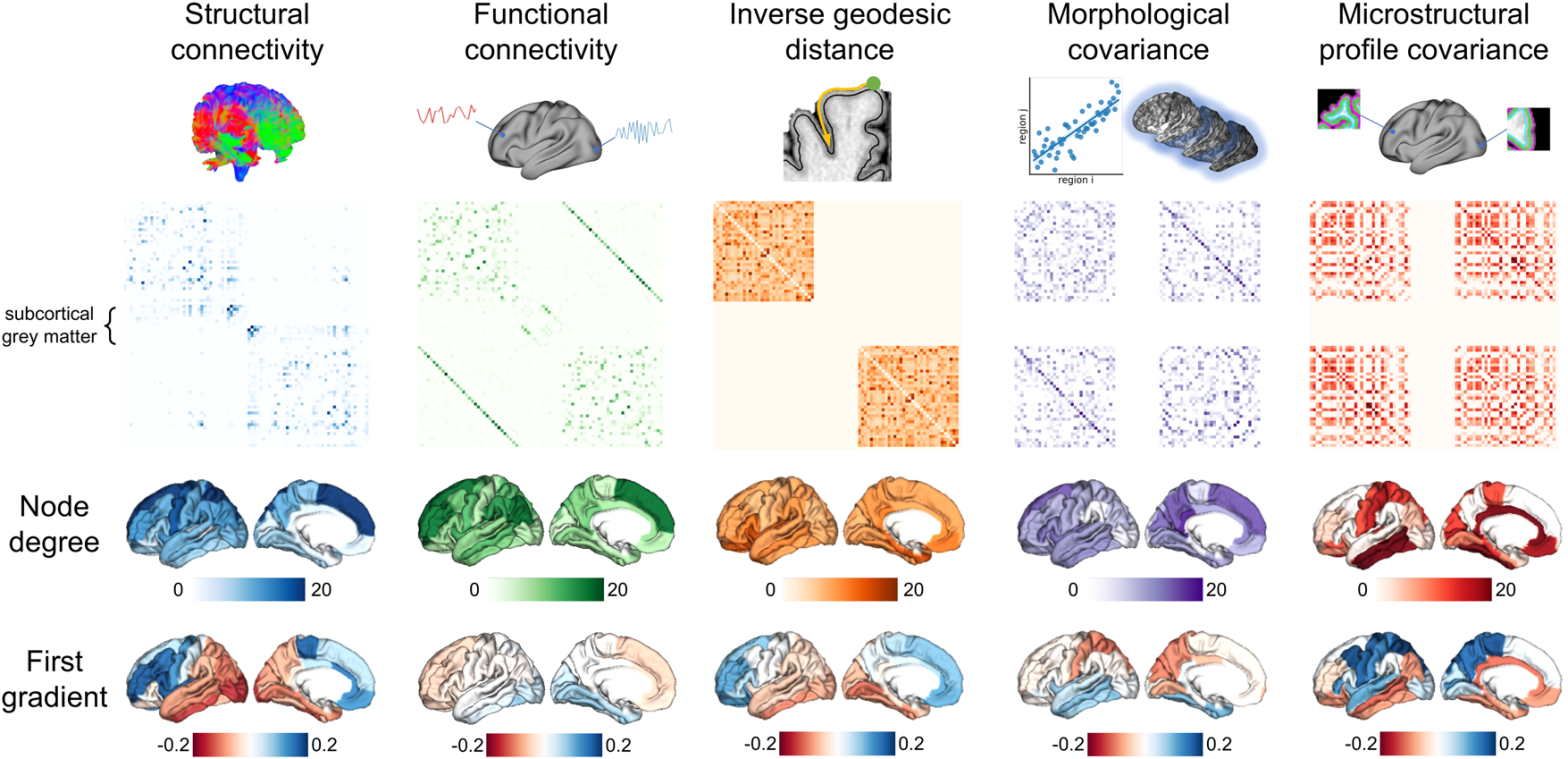
The different methods used to estimate brain connectivity and their corresponding group-averaged connectomes. Note that the inverse geodesic distance, microstructural profile covariance, and morphological covariance networks do not contain any connections to the subcortical grey matter. In addition, the inverse geodesic distance connectome only contains within-hemisphere connections. Node degree is calculated as the number of edges connected to the node at a threshold of 0.1. The gradients were calculated using the methods described in Royer et al. (2022). Node degree and the connectome gradients are displayed on the left hemisphere only for ease of visualisation.

The dataset includes subject-level connectomes, processed using the open-source pipeline, micapipe (https://micapipe.readthedocs.io). A connectome is a symmetric matrix **C**, such that each element C_ij_ describes the connectivity estimate between a pair of regions *i* and *j*. All connectomes were derived in the 68 cortical regions defined by the Desikan-Killiany atlas (Desikan et al., 2006). In addition, the functional and structural connectomes contained subcortical connectivity information from 18 subcortical regions. A visual representation of the group-averaged connectomes is shown in Figure 2, and a brief description of the methods used to generate the connectomes is given below:

##### 2.1.3.1 Tractography derived structural connectivity

Fibre orientations were estimated using constrained spherical deconvolution (Jeurissen et al., 2014). Forty million streamlines were generated using anatomically constrained tractography from MRtrix (Smith et al., 2012; Tournier et al., 2019). The resultant tractograms were filtered with SIFT2, providing each streamline with a weight corresponding to an estimate of its cross-sectional area (Smith et al., 2015). The weighted streamline count was used as the connectivity metric between each region.

##### 2.1.3.2 Functional connectivity

Resting-state fMRI data were mapped to the cortical surface and averaged within each parcel, as well as within each subcortical volumetric region. The partial correlation coefficient of time courses between each pair of regions is used as a connectivity estimate. Partial correlation quantifies the association between the functional activation of two brain regions while conditioning on all other regions, to remove the influence of indirect connections (Marrelec et al., 2006). The partial correlations were calculated using the Pingouin package in python (Vallat, 2018). We used partial correlation instead of full correlation as we found it to greatly improve the model performance (Supplementary Fig. 1).

##### 2.1.3.3 Microstructural profile covariance

Fourteen intracortical surfaces of equal volume were generated to sample the quantitative T1 intensities between the pial surface and the white matter boundary. Vertex-wise intensity profiles were averaged within parcels. Connectivity weights are given by the partial correlation of microstructural profiles between regions, controlling for the average cortex-wide intensity profile. This represents the similarity in the myelin proxies between cortical regions.

##### 2.1.3.4 Inverse geodesic distance

The geodesic distance describes the distance between regions along the cortical mid-thickness mesh. The geodesic distance between the centroid vertex in each parcel and all other vertices on the mid-thickness mesh was calculated using Connectome Workbench (Marcus et al., 2011). The vertex-wise values were averaged within parcels. The inverse distances were used as the connectivity weights, so that nearby regions are more strongly connected.

##### 2.1.3.5 Morphological covariance

We further generated a morphological covariance network from the T1-weighted images in the dataset, as cortical morphology is known to be affected in AD (Lerch et al., 2005; Querbes et al., 2009). The micapipe FreeSurfer processing pipeline was used to calculate the cortical thickness values for each ROI in the Desikan-Killiany atlas (Cruces et al., 2022). Following the methods from He et al. (2007), linear regression was performed at every region to remove the effects of age, gender, age–gender interaction, and mean overall cortical thickness; the residuals of this regression were then substituted for the raw cortical thickness values in a Pearson’s correlation analysis. Connectivity weights are the Pearson’s R value across subjects; regions are considered connected if their cortical thickness covaries across subjects.

All connectomes were averaged across subjects, except morphological covariance, which is calculated at the group level. Negative connectivity weights were set to zero. We compared the performance of the network diffusion model with different thresholds applied to the connectomes, where the threshold values reported describe the proportion of the strongest weights retained. Where present (functional and tractography-derived structural connectomes), subcortical connections were included in the modelling process, although just model predictions from the cortex were retained to match the measured pathology data.

##### 2.1.3.6 Rewired connectomes

We generated 100 randomised connectomes for each modality. These provide a null model to check whether the performance of the models was driven by the empirical connectivity information in the connectomes and could not be achieved by chance with networks that have similar properties. Connectivity weights were iteratively rewired using the Brain Connectivity Toolbox (https://pypi.org/project/bctpy/), with each matrix element rewired approximately ten times. Connectomes are rewired by randomly swapping pairs of edges (e.g., edges connecting nodes A-B and C-D swap to a new configuration A-D and C-B). This approach ensures that the information content of the network is randomised, but nodes never gain or lose an edge, thereby preserving the degree distribution of the network (Váša & Mišić, 2022).

##### 2.1.3.7 Connectome stability analysis

We wanted to determine if a dataset of 50 individuals was sufficient to create a reliable connectome substrate for our model. To do this, we randomly sampled 30 subjects from the dataset, and averaged their connectomes. We repeated this process 100 times, resulting in 100 different connectome samples. We used these connectomes to model tau and atrophy with the network diffusion model and assessed the similarity of the resultant predictions to one another.

##### 2.1.3.8 Connectivity gradients

Connectivity gradients are low-dimensional manifold representations of the connectome, which allow us to visualise the main modes of variation in the multi-dimensional data (Margulies et al., 2016). We calculated the cortical connectivity gradients of the connectome using the BrainSpace toolbox (Vos de Wael et al., 2020), following the methods in Royer et al. (2022). Affinity matrices were generated for each connectome using normalised angle similarity. We applied diffusion map embedding (a non-linear dimensionality reduction technique) to the affinity matrices, yielding a set of gradients ordered by their eigenvalues. We display the first gradient from each modality in Figure 2. These gradients indicate some similarities between the modalities, particularly between the tractography and inverse geodesic distance patterns, but each modality has a unique pattern of variability overall.

### 2.2 Network diffusion model

The network diffusion model was used to model the propagation of neurodegenerative processes through the brain network (Raj et al., 2012). The model assumes diffusive propagation of disease agents between connected brain regions, as described by the equation:

$$\frac{dx\left(t\right)}{dt}=-\beta Hx\left(t\right)$$

where **x**(t) is a vector describing temporal evolution of the pathology signal in each region, β is a diffusivity constant, and **H** is the Laplacian of the connectome **C**, which itself is normalised by the sum of each row to account for differences in region size.

The solution to this equation is given by:

$$x\left(t\right)=e^{-\beta Ht}x_{0}$$

where **x_0_** is the distribution of pathology at t = 0. We used a seeding approach where **x_0_** has a value of one at the (bilateral) seed region and zero elsewhere. We compared results for different seed regions from the Desikan-Killiany atlas. **x**(t) is normalised at each timepoint such that the values lie in the range [0, 1].

As β and t are linearly dependent, we keep β fixed at a constant value and find the optimal timepoint in the model trajectory, by comparing **x** with the measured data at each timepoint t. This procedure is illustrated in Figure 1ii. The optimal timepoint is chosen to be the one that minimises the sum-of-squared error (SSE) between the model prediction and the group-averaged measured data (Fig. 1iii). SSE is less susceptible to local minima than Pearson’s R. The Pearson’s R values between the predicted and observed data at this optimal timepoint (r_max_) are reported for easier interpretation and comparison with other studies.

### 2.3 Combining connectivity estimates to improve model prediction

**Fig. 3.**
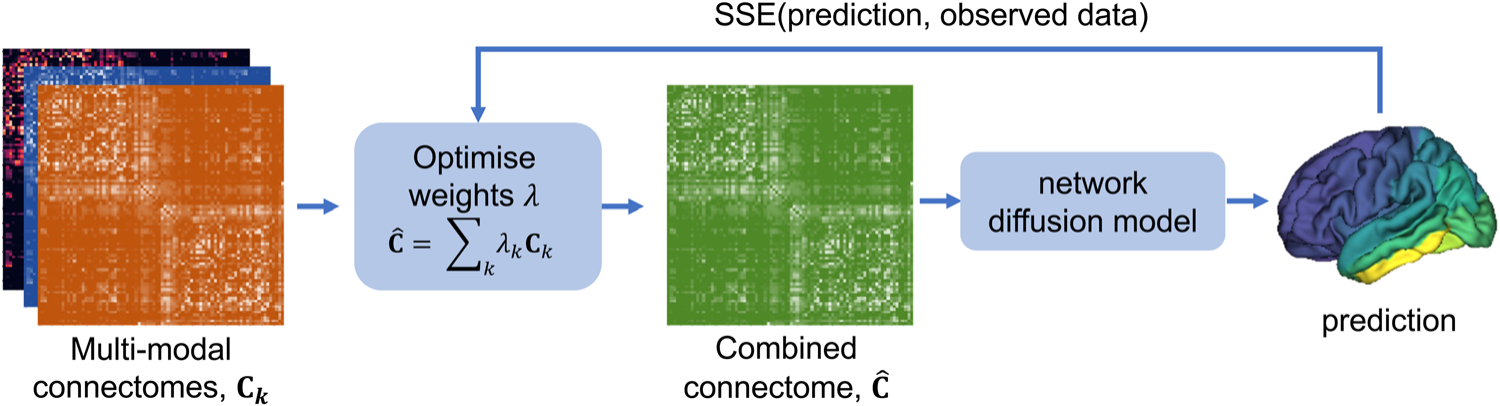
Schematic diagram of the optimisation process. Weights of the connectomes in the linear combination are optimised to yield the output from the network diffusion model that best matches the measured tau PET or atrophy data.

We tested whether using a linear combination of multi-modal connectomes would improve the predictive power of the network diffusion model, compared to using connectomes from the modalities individually. We used a Gaussian-process minimiser (https://scikit-optimize.github.io) to minimise the SSE between the model prediction and the measured data, by optimising the weights $\lambda_{k}$ assigned to each single-modality connectome $C_{k}$ in the combined connectome $\hat{\text{C}}$:

$$\hat{\text{C}}=\sum_{k}\lambda_{k}C_{k}$$

with weights normalised such that $\sum_{k}\lambda_{k}=1$. This process is illustrated in Figure 3. The Gaussian process minimiser was initialised with 300 random starts, before 500 further iterations with the Gaussian process estimator.

**Fig. 4.**
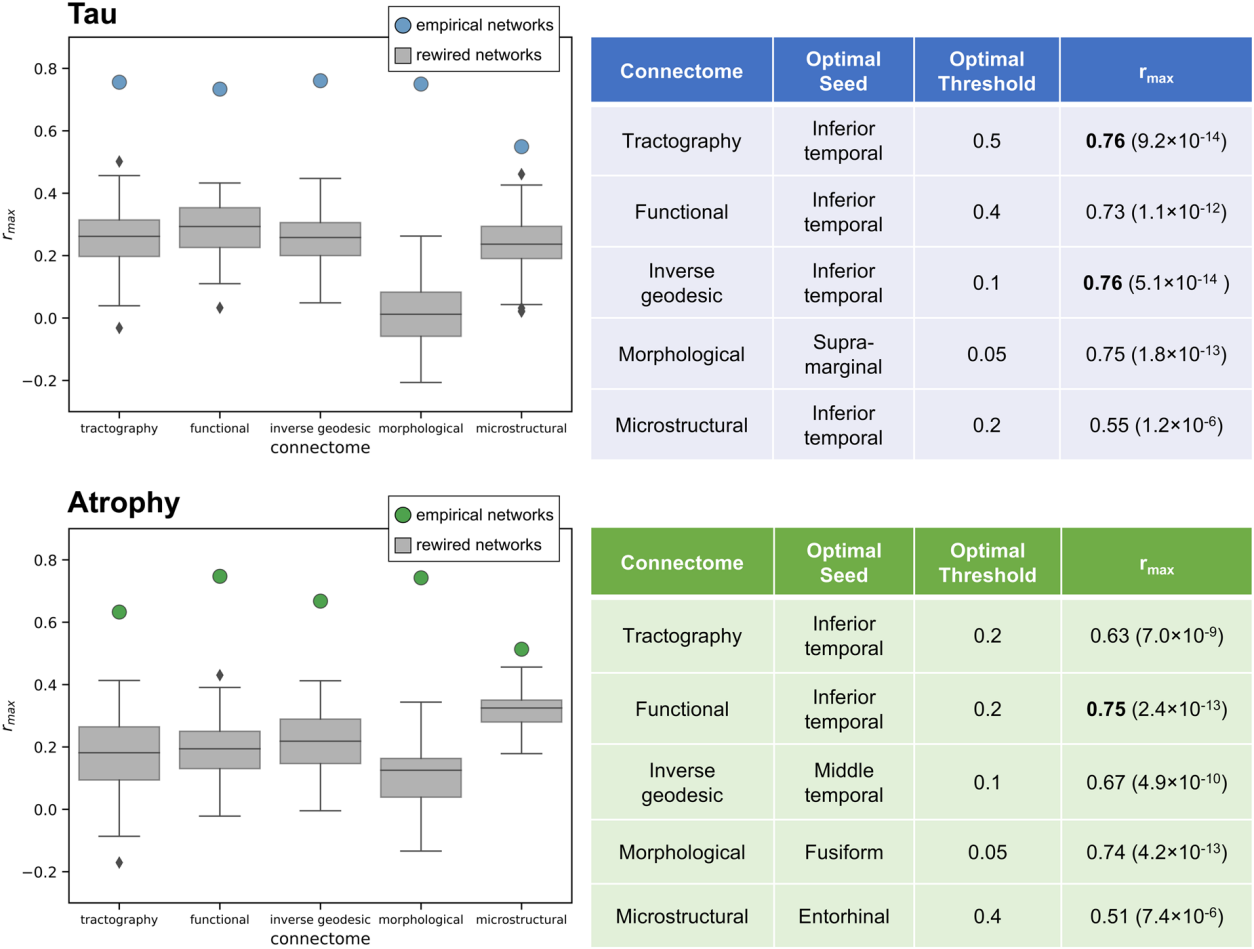
Left: the model performance for each connectome with its best-performing seed region and threshold (circles), compared to the comparable results from a null distribution of 100 rewired networks (boxplots). Performance is measured by r_max_, the Pearson’s correlation between the optimal model prediction and the measured data. Right: Tables displaying the seed region and threshold value that yield the best model agreement for each connectome. The best r_max_ values for each pathology type are highlighted in bold, and p-values of the correlations are displayed in brackets. Results for the group-averaged tau PET data from the ADNI are shown in blue, and the atrophy measurements are shown in green.

The connectomes were thresholded at their optimal values from the single-modality results (given in Fig. 4). The minimisation process was repeated with each of the seed regions that had been shown to provide the most accurate predictions in the single-modality results: entorhinal, fusiform, inferior temporal, middle temporal, and supramarginal regions.

We performed a cross-validation analysis on the ADNI dataset to assess the generalisability of the combined connectome. We randomly divided the tau PET and atrophy datasets into five folds. For each iteration, we used four folds as the training set to optimise the seed and threshold values for each unimodal connectome, as well as the connectome weights and seed for the multimodal connectome. The remaining fold was used as the test set. We ran the network diffusion model using both the best-performing unimodal connectome and the optimised combined connectome from the training set to evaluate the performance on the test set. We were then able to compare the out-of-sample performance of the individual and combined connectomes.

## Results

### 3.1 Different connectomes vary in their ability to support models of neurodegeneration

Figure 4 shows that single-modality connectomes from tractography and inverse geodesic distance are best able to explain patterns of tau deposition via the network diffusion model, and the functional connectome is the best for capturing patterns of cortical atrophy. The morphological connectome also provides good predictions for both types of pathology. All connectomes provided an improved substrate for the network diffusion model compared to the corresponding null distribution of rewired connectomes. This indicates that the results are driven by the empirical connectivity information from the connectomes and cannot be reproduced by randomised networks with similar network properties.

We initialised the model using seed regions from the Desikan-Killiany atlas (results for all thresholds and seed regions are shown in Supplementary Fig. 2). The inferior temporal region provided the best seed region for the model of tau spread, for all but one of the connectomes. The results were more mixed for atrophy; with inferior temporal, entorhinal, middle temporal, and fusiform regions providing the best seeds for the different connectomes. We also tested the performance of each network across a range of different threshold values (0.5, 0.4, 0.3, 0.2, 0.1, and 0.05), which describe the proportion of the strongest weighted connections retained after thresholding. The results in Figure 4 and Supplementary Figure 2 show that the morphological and inverse geodesic networks required a stringent threshold (5-10% of connections retained) for the best model performance.

In Supplementary Figure 3, we explore the stability of our results over a distribution of connectomes sampled from subsets of 30 individuals from the MICA-MICs dataset. Overall, the results demonstrate very high reliability of the model output across connectome samples, except for the morphological network. The morphological network has an average standard deviation in r_max_ of 0.14 across 100 runs, whereas the average standard deviation in r_max_ for the other modalities was less than 0.02. This indicates that a sample size of 50 is sufficient for good model performance for all connectomes except the morphological network.

### 3.2 Combined connectivity information supports more accurate modelling of pathology spread

**Fig. 5.**
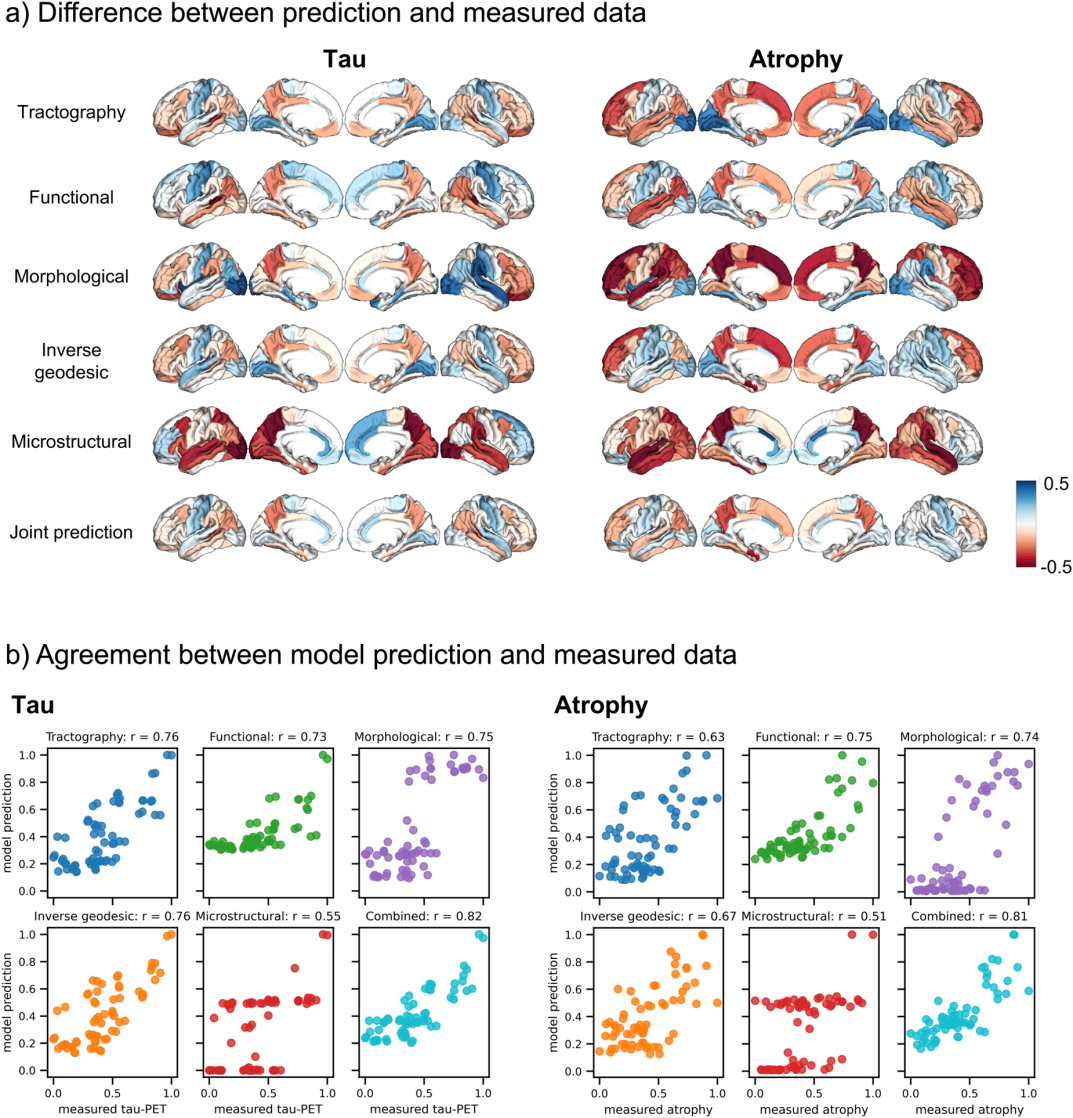
(a) Difference maps: The pathology patterns from the group-averaged data subtracted from the model predictions. Blue areas are those where the model has overpredicted the deposited pathology, red where the model has underpredicted. Overall, the model is more accurate when a joint connectome is used. (b) Scatter plots to illustrate the relationship between the model prediction and measured pathology values for each connectome type. Each point corresponds to a cortical brain region.

Figure 5 shows the agreement between the measured pathology and the optimised model predictions from each of the connectomes. In Figure 5a, we can see that the error between the measured and predicted data is reduced when using the combined connectome, compared to any of the single-modality connectomes. We further show scatter plots with the relationship between the predicted and measured data in Figure 5b, which demonstrate the improvement in model fit when using the combined connectome.

A combination of tractography, morphological, inverse geodesic, and microstructural networks was the best for capturing tau spread (r_max_ = 0.82, p = 1.64 × 10^-17^, inferior temporal seed, weights: 0.61, 0.14, 0.20, 0.05 respectively). For atrophy, the highest r_max_ value of 0.81 (p = 1.29 ×10^-16^) could be achieved with a combination of functional and morphological networks (middle temporal seed, weights: 0.75, 0.25). The predicted tau and atrophy maps are shown in Supplementary Figure 4, alongside their counterparts from the individual modalities.

To confirm that this result was driven by combinations of valid connectivity patterns, we again compared the results with those from rewired networks. Each modality with a non-zero weight in the optimal combination was combined 100 times with randomly chosen rewired connectomes from the other modalities, thresholded at the optimal values, with the weights optimised in the same way as above. The results of this analysis are shown in Supplementary Figure 5. Combining the empirical connectivity information provided a better substrate for the model than could be achieved by adding randomised data to the connectomes.

**Fig. 6.**
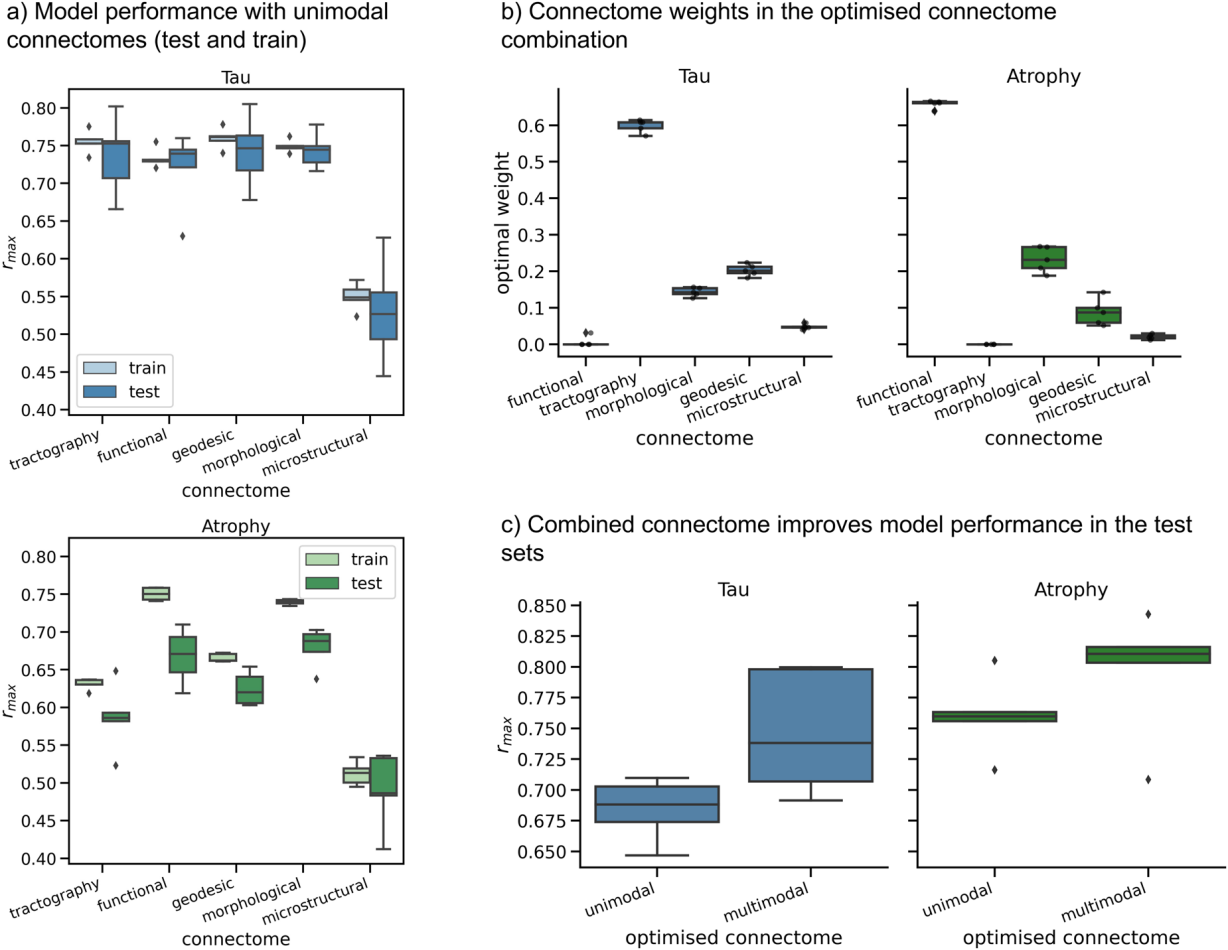
Results from the cross-validation analysis on the ADNI dataset. (a) Boxplots showing the r_max_ values for the individual connectomes in the training and test sets. Seed region and connectome threshold were optimised for the training set in each fold. (b) Weights for each of the constituent modalities in the combined connectome, optimised for each training fold. c) Model performance for the best-performing unimodal connectome in each fold, compared with the optimised combined connectome for that fold. Boxplots show the r_max_ values from the test sets in each fold.

Figure 6 shows the results of the cross-validation analysis. The model performance with unimodal connectomes is more robust for tau PET data than for atrophy, where a greater overall drop in model performance was observed between the training and testing sets (Fig. 6a). Figure 6b shows the weights of the constituent modalities in the combined connectome, as optimised for each training fold. The connectome from tractography was consistently the highest weighted in the combination for modelling tau spread, followed by the inverse geodesic and morphological networks. For atrophy, the functional network was the highest weighted, followed by the morphological network. In Figure 6c, we can see that the combined connectome significantly improved the model performance in the test set, compared to the best-performing individual modality (Wilcoxon signed-rank statistic = 3, p = 0.01). This indicates that the combined connectome is better at explaining patterns of neurodegeneration than single-modality connectomes and is not overfitting to the data that are used to find the weights.

### 3.3 Combined connectome improves modelling of neurodegeneration in unseen datasets

**Fig. 7.**
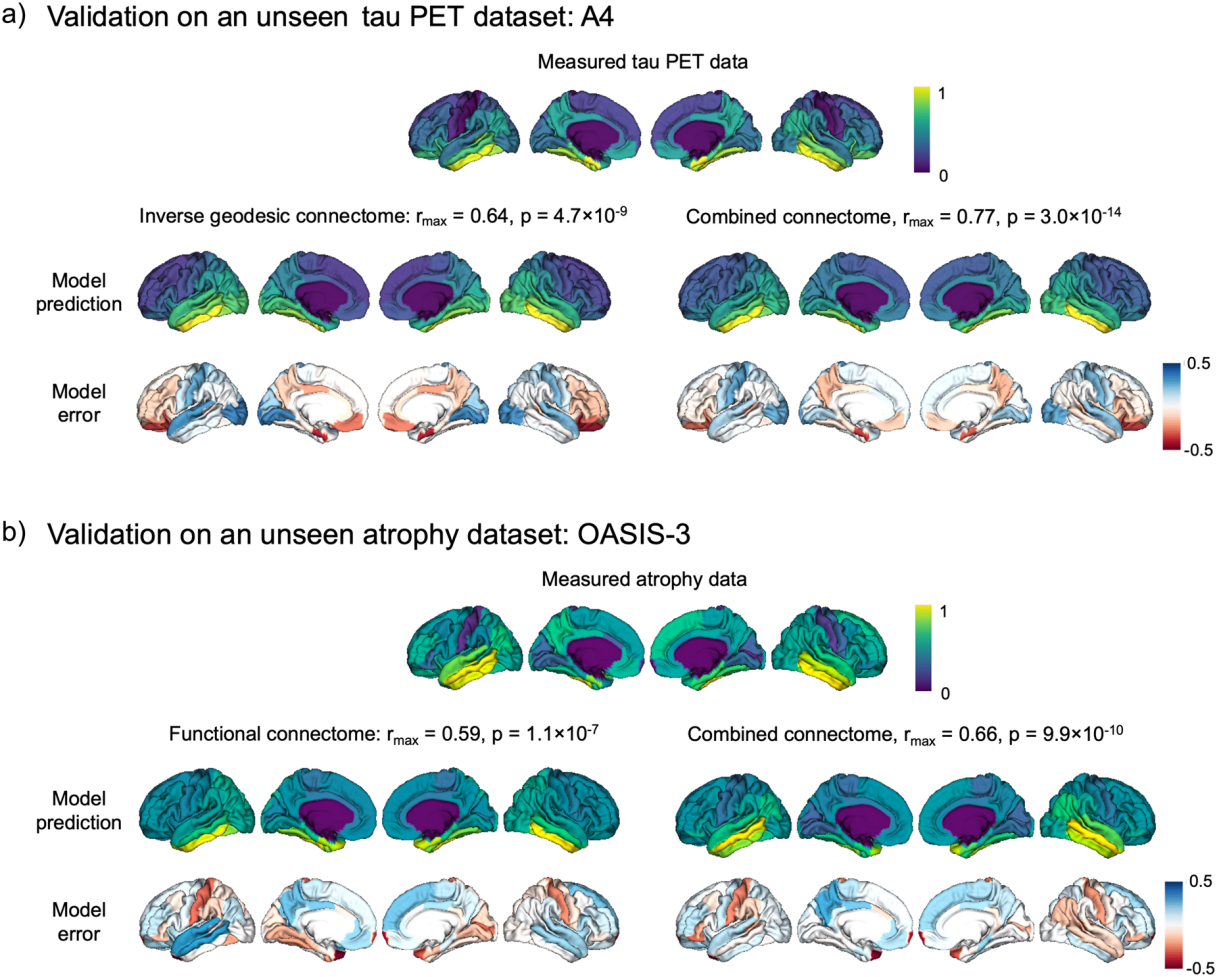
Results from the application of our combined connectome model to external datasets. (a) Validation in tau PET data from cognitively unimpaired individuals from the A4 study. Top row: the average tau PET SUVRs from the “elevated tau” group (see methods). Middle row: the model predictions from the inverse geodesic connectome and the prediction from the combined connectome, with seed region and connectome weights optimised for the ADNI dataset. Bottom row: difference maps between the predicted and measured values. Regions in blue are overpredicted by the model, regions in red are underpredicted. (b) Validation in atrophy data from cognitively impaired individuals from OASIS-3. Top row: the average atrophy pattern. Middle row: the model predictions from the functional connectome and the prediction from the combined connectome, with seed region and connectome weights optimised for the ADNI dataset. Bottom row: difference maps between the predicted and measured values.

We tested the combined connectome on two further tau PET datasets, comprising cognitively unimpaired individuals from ADNI and the A4 study, both with elevated tau PET status (Sperling et al., 2014). Using the same thresholds and connectome weights that had been optimised for the cognitively impaired ADNI dataset, we compared the predictive performance of the model using the best-performing unimodal connectome (inverse geodesic), and the combined connectome. In both cases, we used the inferior temporal region as the seed region. The results in Figure 7a illustrate the improvement in model performance using the combined connectome for modelling the data from the A4 study. This increase in r_max_ from 0.64 to 0.77 was deemed to be significant according to Hotelling’s t-test (t = -2.4, p = 0.02). The combined connectome also significantly improved model performance for the cognitively unimpaired ADNI subjects, from r_max_ = 0.73 with the inverse geodesic network to r_max_ = 0.85 with the combined connectome (t = -2.9, p = 0.005). The results from this subset of the ADNI dataset are shown in Supplementary Figure 6.

We then used regional cortical volume measures from the OASIS-3 dataset to test whether the combined connectome improved model prediction of atrophy in an external cohort of cognitively impaired individuals. We again compared the unimodal connectome that performed best on the ADNI dataset (functional connectome, with inferior temporal seed), with the connectome combination that gave the best model prediction in ADNI (combined functional and morphological connectome, middle temporal seed). The results of this analysis are shown in Figure 7b. The combined connectome yielded an improvement in model performance (from r_max_ = 0.59 to r_max_ = 0.66), but this did not reach significance (Hotelling’s t = -1.3, p = 0.19).

## 4 Discussion

In this study, we compared the ability of the network diffusion model to capture tau and atrophy spread in AD using connectomes from five different imaging modalities. We found that combining information from different connectomes can further improve the model fit, for predicting both tau and atrophy patterns (see Fig. 5). The highest levels of model discrepancy from the individual connectomes were reduced; however, the combined connectome still demonstrates some overprediction of pathology in the sensorimotor areas, and underprediction in the inferior parietal lobe and in the precuneus. This shows that combinations of connectomes can better replicate disease pathology patterns when used as a substrate for progression modelling than connectomes from any individual modality. This supports findings from recent work where combining estimates of brain connectivity from different modalities provides increased sensitivity to changes related to neurodegenerative disease (Fedorov et al., 2021; L. Zhang et al., 2021; Y. Zhang et al., 2019). Our results might indicate that the different modalities provide complementary information that can be combined to give a more accurate picture of the brain’s connectome. Alternatively, this can be thought of in an analogous way to ensemble modelling, where biases inherent to each modality cancel out to provide a more accurate prediction.

We tested the ability of the combined connectome to model tau PET data from cognitively unimpaired individuals from both the ADNI and A4 studies. We found it necessary to have elevated tau PET as an inclusion criterion for these groups, to ensure that there is sufficient disease signal in the tau PET data for the model prediction. Despite being cognitively unimpaired, these individuals are situated in the AD continuum and are therefore at a higher risk of developing dementia due to their brain amyloid levels. This analysis used the combined connectome derived from the original cognitively impaired tau PET group, to test its generalisability to other cohorts and disease stages. We observed an overall lower model performance for the A4 data in comparison to the data from ADNI. This indicates that the model is sensitive to cohort effects. However, we saw a significant improvement in model performance for both groups when using the multimodal connectome, compared to the inverse geodesic connectome. The advantage of the combined connectome for modelling tau is therefore not specific to the ADNI cohort or disease stage. We were unable to validate the atrophy model in these cognitively unimpaired datasets due to the lack of disease signal in the brain volume data. We did however test the combined connectome for modelling atrophy spread in an external dataset of individuals with MCI and dementia, from the OASIS-3 study. Although the combination of functional and morphological connectomes optimised for atrophy modelling in ADNI provided some improvement in model fit to the OASIS dataset, the improvement was not statistically significant. This could be due to the different levels of cognitive impairment in the datasets (see Table 2) and suggests that the optimised combined connectome is more robust for modelling tau propagation than atrophy. We have not investigated whether the same combination of connectomes emerges when deriving the model in individuals with varying degrees of cognitive impairment. Future work could explore how the relative weightings of connectomes within the combined connectome change across disease stages.

When using single-modality connectomes, we found that connectomes from tractography and inverse geodesic distance were best able to support models of tau pathology spread. The good performance of the tractography connectome supports the transsynaptic spread hypothesis, in line with results from previous work (Raj et al., 2012; Vogel et al., 2020). Our results indicate that the tractography and inverse geodesic connectomes contain overlapping information: their primary gradients follow a similar anterior-posterior pattern (Fig. 2), and their model predictions are qualitatively similar (Supplementary Fig. 4). This aligns with results from previous work that has demonstrated that the connectivity weight measured between two regions with tractography scales inversely with the distance between them (Donahue et al., 2016). The optimal seed region for modelling tau spread was found to be the inferior temporal region, for both unimodal and multimodal connectomes (except for the morphological network). This contradicts the commonly accepted idea that tau originates in the transentorhinal cortex (Braak & Braak, 1991), suggesting that the network diffusion model lacks the complexity to accurately extrapolate backwards to an earlier, unseen stage of the disease. Rather, this result can be interpreted as the model identifying the inferior temporal region as a hub at the stage of the disease sampled in this cohort, which agrees with previous work (Lee et al., 2022). As well as connectome weightings, future work could explore if the optimal seed regions change at different disease stages.

Although not included in the original MICA-MICs dataset, morphological covariance networks were included in this study because of the wealth of literature relating them to AD pathology. Our results show that the morphological network is one of the most effective networks for modelling both tau and atrophy. Morphological networks exhibit both similarities and divergences from structural connectomes (Gong et al., 2012) and functional connectivity networks (Alexander-Bloch, Raznahan, et al., 2013), which suggests that the networks share some of the same underlying processes but that the morphological network contains different information to the other modalities. This is supported by our results in Figure 6b that show that the morphological network is a key component of the combined connectomes. However, we also found that the morphological network is less reliable than the other networks considered in our analyses (Supplementary Fig. 3). This is a limitation of the current study; our results indicate that a larger sample size is needed to generate a morphological connectome that provides stable predictions from the network diffusion model. In the morphological network, connectivity weights are assigned to pairs of regions based on the covariance in their thickness across individuals. Multiple interpretations have been proposed for this covariance, including shared developmental factors (Vértes & Bullmore, 2015); experience-related plasticity (Alexander-Bloch, Giedd, et al., 2013); and gene expression (Romero-Garcia et al., 2018). Shafiei et al. (2022) showed that incorporating gene expression into a model of pathology propagation in frontotemporal dementia improved the models’ ability to capture atrophy patterns. Future work could help determine whether the unique contributions of the morphological network to our model are linked to genetic factors or other features.

The functional connectome facilitated the most accurate model of atrophy and was a key component of the combined connectome optimised for atrophy modelling. As a preliminary experiment, we compared the performance of the network diffusion model when using full and partial correlations between functional time courses as our measure of functional connectivity. We found that model performance across all seeds and connectome thresholds was much improved when using partial correlations as the connectome edge weights, as shown in Supplementary Figure 1. This demonstrates the advantage of partial correlation as a denoising approach when estimating functional connectivity (Marrelec et al., 2006).

One drawback of this work is the use of a simple version of the network diffusion model with one tuneable parameter. This model was chosen because it is parsimonious but is still able to capture the measured patterns of pathology. However, more complex models such as the epidemic spreading model (Iturria-Medina et al., 2014; Vogel et al., 2020), kinetic models (Fornari et al., 2019; Weickenmeier et al., 2019), and the S-I-R model (Shafiei et al., 2022) may be able to provide more biologically interpretable parameters. In future work, it would be interesting to see if the results shown here can be replicated with these other models. Furthermore, we only use a single model timepoint for the prediction, which neglects the full temporal state of the model. Future work could also utilise the full model trajectory to evaluate the temporal evolution of the disease process against longitudinal data.

Throughout this work, we used the Desikan-Killiany atlas, which has been very commonly used in the field of network-spreading models (Montal et al., 2022; Schäfer et al., 2020; Vogel et al., 2020; Yang et al., 2021; Zheng et al., 2019). However, this atlas omits several crucial brain regions in AD, such as the grey matter of the hippocampus. The off-target binding of the tau PET tracer in the choroid plexus affects nearby subcortical structures, including the hippocampus, so we remove these regions from our analysis (Lemoine et al., 2018). With the development of new PET tracers and techniques for mitigating the effects of off-target binding, in future we hope to be able to incorporate these regions into our models, which would necessitate the use of a different atlas.

A further limitation of our framework is that it does not consider the heterogeneity present in both brain connectivity and pathology patterns in AD. We used averaged connectomes from young, healthy adult subjects as the substrate for our model, as datasets including both high-quality multimodal connectomics data and pathology measures in older individuals are limited. However, degenerative processes associated with AD might impact brain networks in such a way that would modify the disease-spreading process. In addition, it has been demonstrated that there are important inter-individual differences in brain networks (Finn et al., 2015; Mansour et al., 2021), which are neglected by our template-based approach. The literature is mixed on the benefits of patient-specific connectomes versus template connectomes for the prediction of tau accumulation. Patient-specific approaches have been shown to improve model accuracy (Brown et al., 2019; Franzmeier et al., 2020; Leuzy et al., 2023). However, a study by Powell et al. (2018) showed that the use of patient-specific connectomes does not improve prediction of a patient’s future grey matter atrophy in the network diffusion model, compared to an age-matched, control connectome. Furthermore, Vogel et al. (2020) found that structural connectomes from tractography in young healthy subjects were best able to model tau spread in the epidemic spreading model, when compared to connectomes from older individuals in the ADNI dataset. An interesting avenue for future work would be to test our combined connectome framework for personalised pathology predictions, and to see whether it can provide insight into the inter-individual variability observed in tau PET and atrophy patterns (Poulakis et al., 2018; Vogel et al., 2021, 2023). Furthermore, the upcoming UK Biobank connectome dataset will be a valuable resource for generating template connectomes from older individuals, which could be used to improve the performance of our models (Mansour et al., 2023).

Many other methods for connectome aggregation have been used in the literature outside of the field of network-spreading models, for example graphical neural networks (L. Zhang et al., 2021; Y. Zhang et al., 2023) and multi-layer networks (Battiston et al., 2018; Casas-Roma et al., 2022). While our weighted sum approach is simple compared to these methods, the interpretability of the individual connectome weights is an advantage. Within the field of connectome-based pathology modelling, functional and structural connectivity information have previously been combined by Zheng et al. (2019), using the S-I-R model to capture patterns of alpha-synuclein in Parkinson’s disease. They demonstrated improved model performance when weighting the edges of the structural connectome by an exponential functional connectivity factor. However, to the best of our knowledge, our work is the first attempt at task-driven aggregation of connectomes, and the first to combine more than two different connectivity modalities in a data-driven way.

## Supplementary Material

Supplementary Material

## Data Availability

Data from ADNI and the A4 study are publicly and freely available from the Laboratory of Neuro Imaging (LONI) Image and Data Archive upon sending a request that includes the proposed analysis and the named lead investigator, at http://adni.loni.usc.edu/data-samples/access-data/and https://ida.loni.usc.edu/home/projectPage.jsp?project=A4, respectively. OASIS-3 is an open-access dataset which can be accessed at https://www.oasis-brains.org. MICA-MICs is available on the Canadian Open Neuroscience Platform data portal (https://portal.conp.ca) and the Open Science Framework (https://osf.io/j532r/). Python code for running the network diffusion model and optimising a combined connectome can be found at: https://github.com/ethompson93/multimodal_ndm.

## 5 Conclusions

In summary, we have demonstrated that combining information from different modalities into a single connectome improves our ability to explain pathology patterns with the network diffusion model, compared to any modality individually. We systematically characterised the connectome-specific performance of the network diffusion model across five different connectomes to reveal new insight into the properties of the connectomes and their ability to capture patterns of neurodegeneration in AD. Our results suggest that combining the unique features from different modalities can mitigate some of the modality-specific biases and provide a better substrate for modelling the trajectories of pathology spread in AD.
