# Supplementary material for: Combining multimodal connectivity information improves modelling of pathology spread in Alzheimer’s disease
Source: Imaging Neurosci (Camb). 2024 Feb 5;2:imag-2-00089. doi: 10.1162/imag_a_00089 (PMC11211996; doi:10.1162/imag_a_00089)
Supplement: Supplementary Material [file imag_a_00089-supp.pdf]

Combining Multimodal Connectivity Information Improves Modelling of Pathology Spread in Alzheimer's Disease

Supplementary Material

Elinor Thompson[1], Anna Schroder[1], Tiantian He[1], Cameron Shand[1], Sonja Soskic[2], Neil P. Oxtoby[1], Frederik Barkhof[1,3,4], Daniel C. Alexander[1] for the Alzheimer's Disease Neuroimaging Initiative[†]*

1. *UCL Centre for Medical Image Computing, Department of Computer Science, University College London, London, UK*
2. *UCL Centre for Medical Image Computing, Department of Medical Physics and Biomedical Engineering, University College London, London, UK*
3. *Department of Radiology & Nuclear Medicine, Amsterdam UMC, Vrije Universiteit, the Netherlands*
4. *UCL Queen Square Institute of Neurology, University College London, UK*

**Corresponding author: Dr Elinor Thompson*
Email: elinor.thompson@ucl.ac.uk

[†] *Data used in preparation of this article were obtained from the Alzheimer's Disease Neuroimaging Initiative (ADNI) database (adni.loni.usc.edu). As such, the investigators within the ADNI contributed to the design and implementation of ADNI and/or provided data but did not participate in analysis or writing of this report. A complete listing of ADNI investigators can be found at: http://adni.loni.usc.edu/wp-content/uploads/how_to_apply/ADNI_Acknowledgement_List.pdf*

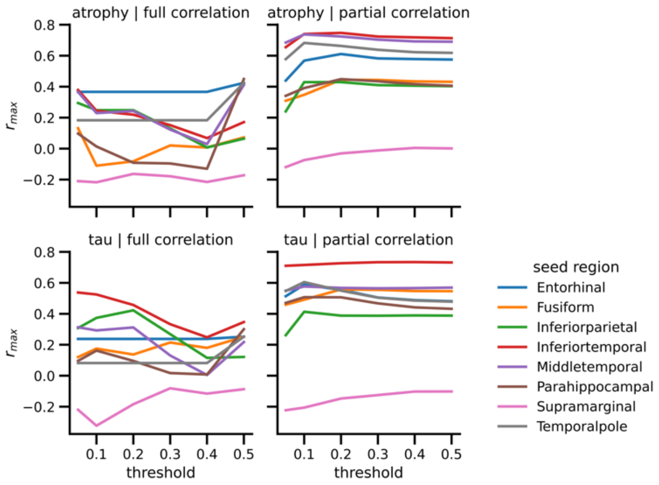

Supplementary figure 1. Comparing the performance of the network diffusion model using connectomes derived from two types of functional connectivity: full correlation (left column) and partial correlation (right column). Model agreement with the measured data (r_{max}), is shown for different seed regions and connectome threshold values. Model performance for atrophy is shown on the top row, and tau-PET is shown on the bottom row.

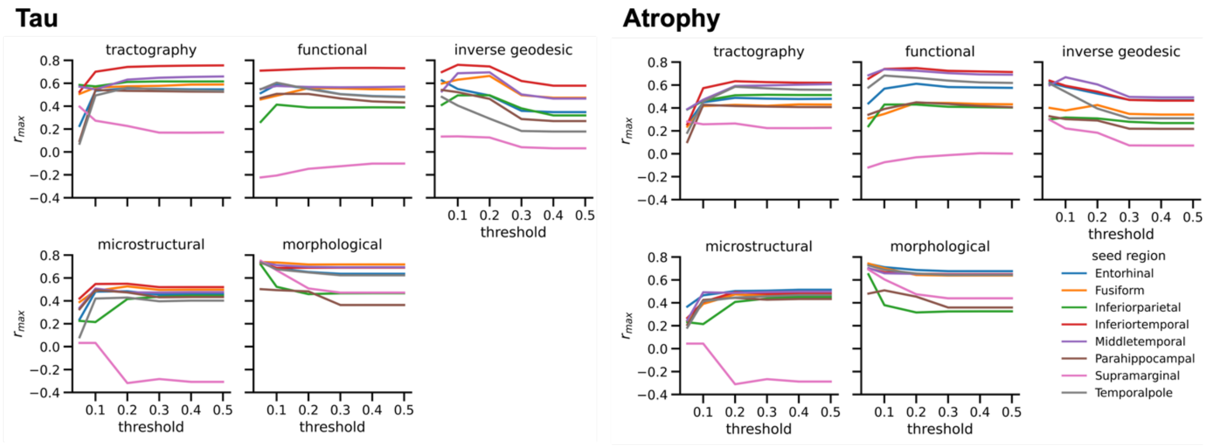

Supplementary figure 2. The results of the network diffusion model across different input connectomes, different connectome thresholds, and different seed regions. Performance is measured by r_{max}, the correlation between the optimal model prediction and the group-averaged tau-PET or atrophy pattern. Only the best eight seed regions are shown for ease of visualisation. Threshold values describe the proportion of connections retained.

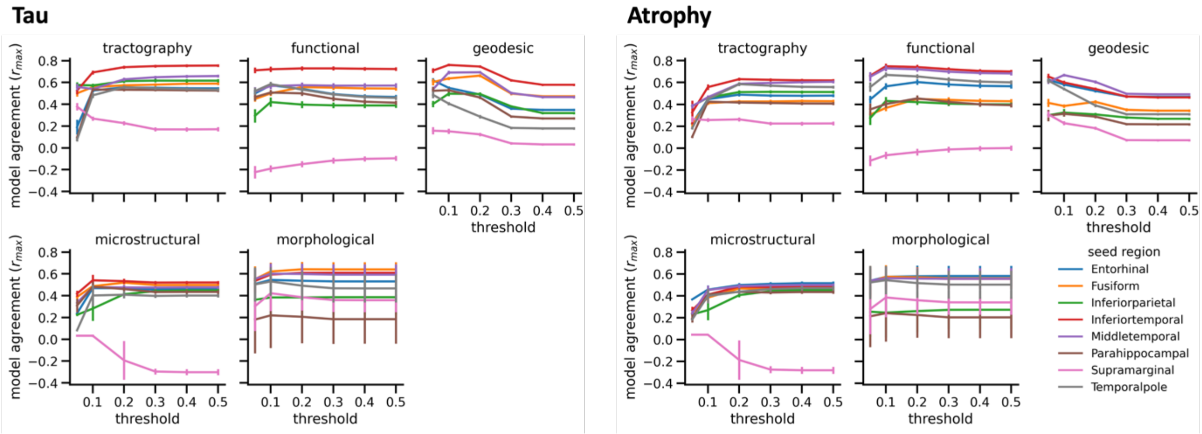

Supplementary figure 3. Results from the connectome stability analysis. Error bars show the standard deviation across 100 sampled connectomes, each comprised of data from 30 individuals randomly sampled from the total group of 50 healthy controls. The model was repeated 100 times for each modality, at different connectome thresholds, and with different seed regions. Performance is measured by r_{max}, the correlation between the optimal model prediction and the group-averaged tau-PET or atrophy pattern. Only the best eight seed regions are shown for ease of visualisation.

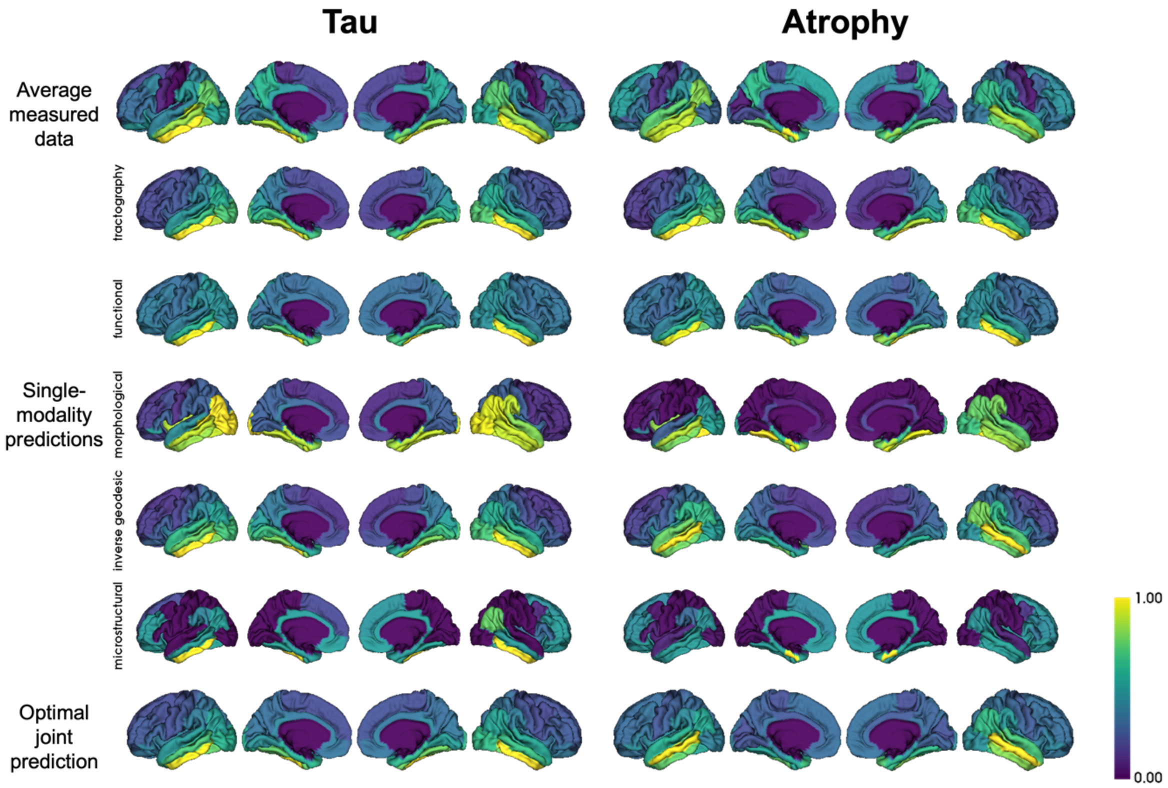

Supplementary figure 4. Group averaged tau-PET and atrophy data compared visually with the best model predictions from the individual connectomes and the optimal connectome combination.

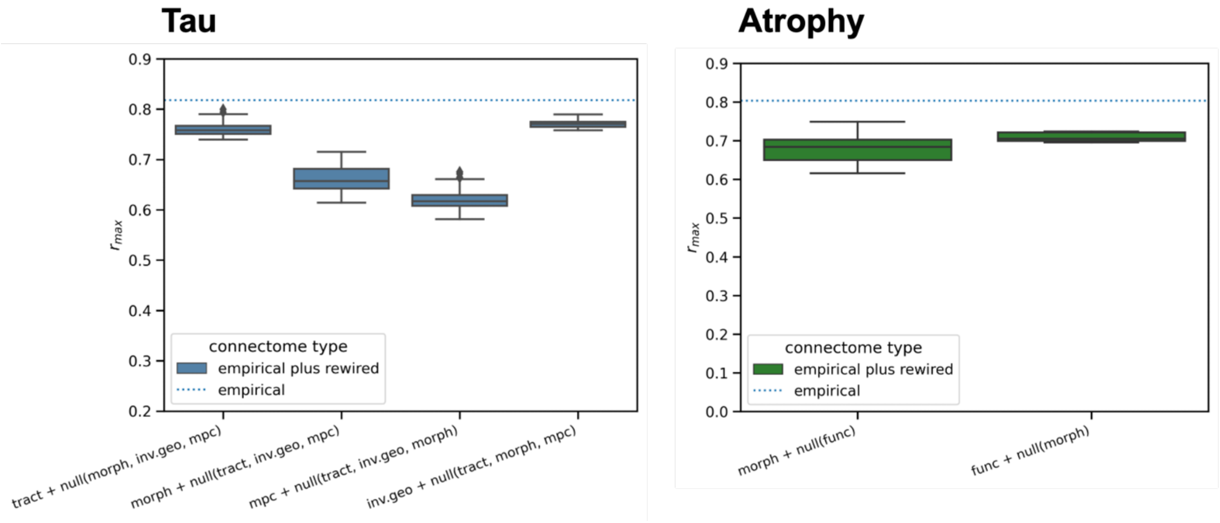

Supplementary figure 5. The best results from the combined connectomes (blue dotted lines) were compared against 100 random combinations of each constituent connectome and rewired connectomes from the other modalities in the set (boxplots). In each case, the combined empirical connectomes provided a better substrate for the model than a combination of rewired and empirical connectomes, as indicated by the higher r_{max} value of the dotted line, compared to the boxplots.

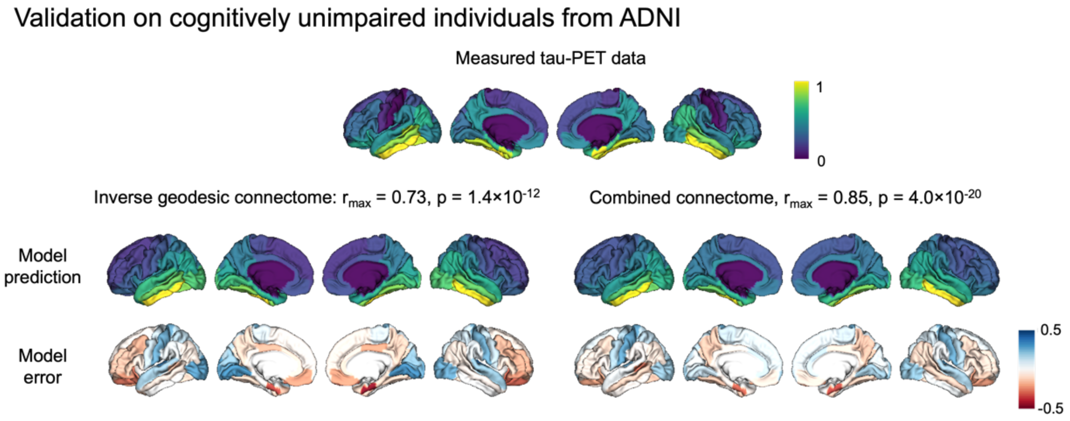

Supplementary figure 6. Model predictions of tau-PET data from cognitively unimpaired individuals from ADNI. Top row: the average tau-PET SUVR pattern. Middle row: the model predictions from the inverse geodesic connectome and the prediction from the combined connectome, with seed region and connectome weights optimised for the cognitively impaired ADNI dataset. Bottom row: difference maps between the predicted and measured values. Regions in blue are overpredicted by the model, regions in red are underpredicted.
